# Supplementary material for: Hot spot prediction in protein-protein interactions by an ensemble system
Source: BMC Syst Biol. 2018 Dec 31;12(Suppl 9):132. doi: 10.1186/s12918-018-0665-8 (PMC6311905; doi:10.1186/s12918-018-0665-8)
Supplement: Supplementary file 2 — All relevant MATLAB code. A simple Matlab implement of our predictor. (ZIP 19,742 kb) [file 12918_2018_665_MOESM2_ESM.zip › Additional file 2/Read ASA/ASA website/SKEMPI/1CBWI.htm]

 NetSurfP - Protein Surface Accessibility and Secondary Structure Predictions

  

|  |  |  |
| --- | --- | --- |
|  |  | NetSurfP - Protein Surface Accessibility and Secondary Structure Predictions  Technical University of Denmark |

  


---

```
# For publication of results, please cite:
# A generic method for assignment of reliability scores applied to solvent accessibility predictions.
# Bent Petersen, Thomas Nordahl Petersen, Pernille Andersen, Morten Nielsen and Claus Lundegaard
# BMC Structural Biology 2009, 9:51 doi:10.1186/1472-6807-9-51
#
# Column 1: Class assignment - B for buried or E for Exposed - Threshold: 25% exposure, but not based on RSA
# Column 2: Amino acid
# Column 3: Sequence name
# Column 4: Amino acid number
# Column 5: Relative Surface Accessibility - RSA
# Column 6: Absolute Surface Accessibility
# Column 7: Z-fit score for RSA prediction
# Column 8: Probability for Alpha-Helix
# Column 9: Probability for Beta-strand
# Column 10: Probability for Coil
E R  1CBWI                  1    0.763 174.773   1.102   0.003   0.003   0.994
E P  1CBWI                  2    0.488  69.190  -0.174   0.018   0.019   0.964
E D  1CBWI                  3    0.621  89.515  -0.896   0.052   0.084   0.864
E F  1CBWI                  4    0.367  73.717   1.245   0.052   0.084   0.864
B C  1CBWI                  5    0.076  10.614  -2.060   0.052   0.084   0.864
E L  1CBWI                  6    0.534  97.775   0.027   0.019   0.141   0.840
B E  1CBWI                  7    0.314  54.803  -0.211   0.018   0.088   0.893
E P  1CBWI                  8    0.424  60.222   0.603   0.018   0.088   0.893
E P  1CBWI                  9    0.515  73.050  -0.812   0.018   0.047   0.935
E Y  1CBWI                 10    0.522 111.616  -0.793   0.018   0.088   0.893
E T  1CBWI                 11    0.569  78.976  -0.066   0.018   0.088   0.893
B G  1CBWI                 12    0.149  11.687  -2.983   0.019   0.141   0.840
E P  1CBWI                 13    0.492  69.786   0.603   0.019   0.141   0.840
B C  1CBWI                 14    0.046   6.486  -2.470   0.052   0.084   0.864
E K  1CBWI                 15    0.485  99.826   0.444   0.052   0.084   0.864
E A  1CBWI                 16    0.445  49.006  -1.240   0.056   0.142   0.802
E R  1CBWI                 17    0.373  85.486  -0.144   0.021   0.279   0.699
B I  1CBWI                 18    0.177  32.726   0.055   0.021   0.451   0.528
B I  1CBWI                 19    0.259  47.897   0.140   0.023   0.655   0.322
B R  1CBWI                 20    0.114  26.220   0.421   0.018   0.846   0.136
B Y  1CBWI                 21    0.083  17.652   0.193   0.011   0.918   0.071
B F  1CBWI                 22    0.102  20.431  -0.244   0.011   0.918   0.071
B Y  1CBWI                 23    0.071  15.258   0.296   0.018   0.846   0.136
B N  1CBWI                 24    0.168  24.595  -0.105   0.022   0.552   0.426
B A  1CBWI                 25    0.312  34.393  -0.176   0.018   0.088   0.893
E K  1CBWI                 26    0.656 134.877   0.823   0.018   0.047   0.935
E A  1CBWI                 27    0.511  56.257   0.096   0.018   0.047   0.935
E G  1CBWI                 28    0.451  35.533   0.351   0.018   0.047   0.935
E L  1CBWI                 29    0.411  75.272   1.917   0.004   0.514   0.481
B C  1CBWI                 30    0.043   6.009   0.155   0.002   0.816   0.182
B Q  1CBWI                 31    0.269  48.079   0.859   0.001   0.900   0.099
E T  1CBWI                 32    0.353  48.906   0.567   0.001   0.900   0.099
B F  1CBWI                 33    0.054  10.778   0.823   0.011   0.918   0.071
B V  1CBWI                 34    0.278  42.652  -0.142   0.001   0.900   0.099
B Y  1CBWI                 35    0.125  26.712  -0.782   0.003   0.718   0.279
B G  1CBWI                 36    0.177  13.914  -2.296   0.021   0.451   0.528
B G  1CBWI                 37    0.220  17.298  -1.834   0.021   0.279   0.699
B C  1CBWI                 38    0.158  22.239  -1.704   0.020   0.205   0.775
E R  1CBWI                 39    0.336  76.921  -0.938   0.018   0.088   0.893
E A  1CBWI                 40    0.432  47.584  -2.072   0.018   0.019   0.964
E K  1CBWI                 41    0.305  62.800  -0.609   0.018   0.019   0.964
E R  1CBWI                 42    0.576 131.881  -0.205   0.058   0.017   0.925
B N  1CBWI                 43    0.239  34.916  -0.897   0.058   0.017   0.925
E N  1CBWI                 44    0.357  52.206   0.166   0.115   0.016   0.868
B F  1CBWI                 45    0.151  30.406  -0.898   0.339   0.016   0.645
E K  1CBWI                 46    0.530 109.042   0.220   0.257   0.016   0.727
E S  1CBWI                 47    0.210  24.565   0.331   0.181   0.016   0.803
E A  1CBWI                 48    0.266  29.291   1.171   0.923   0.002   0.076
E E  1CBWI                 49    0.632 110.480   1.333   0.970   0.001   0.030
E D  1CBWI                 50    0.413  59.470   2.266   0.970   0.001   0.030
B C  1CBWI                 51    0.014   1.938   1.242   0.988   0.000   0.012
E M  1CBWI                 52    0.381  76.198   1.902   0.970   0.001   0.030
E R  1CBWI                 53    0.668 152.857   1.588   0.938   0.007   0.055
E T  1CBWI                 54    0.362  50.168   1.887   0.938   0.007   0.055
B C  1CBWI                 55    0.062   8.747  -1.375   0.782   0.003   0.216
E G  1CBWI                 56    0.609  47.928   0.592   0.406   0.004   0.590
E G  1CBWI                 57    0.691  54.342  -0.705   0.176   0.004   0.820
E A  1CBWI                 58    0.725  79.862  -0.693   0.003   0.003   0.994

Explain the output.  Go back.
```

---
